# The role of extracelluar matrix in osteosarcoma progression and metastasis

**DOI:** 10.1186/s13046-020-01685-w

**Published:** 2020-09-04

**Authors:** Juncheng Cui, Dylan Dean, Francis J. Hornicek, Zhiwei Chen, Zhenfeng Duan

**Affiliations:** 1 Department of Orthopedic Surgery, The First Affiliated Hospital of University of South China, 69 Chuanshan Road, Hengyang, 421001 Hunan China; 2 Department of Orthopedic Surgery, Sarcoma Biology Laboratory, David Geffen School of Medicine at UCLA, 615 Charles E. Young Dr. South, Los Angeles, CA 90095 USA

**Keywords:** Extracellular matrix, Osteosarcoma, Metastasis, Prognostic biomarker, Therapeutic target

## Abstract

Osteosarcoma (OS) is the most common primary bone malignancy and responsible for considerable morbidity and mortality due to its high rates of pulmonary metastasis. Although neoadjuvant chemotherapy has improved 5-year survival rates for patients with localized OS from 20% to over 65%, outcomes for those with metastasis remain dismal. In addition, therapeutic regimens have not significantly improved patient outcomes over the past four decades, and metastases remains a primary cause of death and obstacle in curative therapy. These limitations in care have given rise to numerous works focused on mechanisms and novel targets of OS pathogenesis, including tumor niche factors. OS is notable for its hallmark production of rich extracellular matrix (ECM) of osteoid that goes beyond simple physiological growth support. The aberrant signaling and structural components of the ECM are rich promoters of OS development, and very recent works have shown the specific pathogenic phenotypes induced by these macromolecules. Here we summarize the current developments outlining how the ECM contributes to OS progression and metastasis with supporting mechanisms. We also illustrate the potential of tumorigenic ECM elements as prognostic biomarkers and therapeutic targets in the evolving clinical management of OS.

## Background

Osteosarcoma (OS) is the most common primary bone malignancy and disproportionately affects those in childhood and adolescence [1]. Before the widespread use of chemotherapy in the 1970s, surgical resection was the primary treatment modality available to OS patients [2]. Adjuvant chemotherapy has since dramatically improved the prognosis for OS patients, with the five-year survival rate increased from 20% to approximately 55 to 70% in patients with localized disease [3, 4]. However, in cases of metastatic lesions, the five-year survival rate remains dismal at less than 20% [5]. Targeting and preventing metastasis has thus been a significant obstacle in OS treatment, and recent publications have highlighted various novel treatment strategies to that end. The dysregulation and aberrant remodeling of extracellular matrix (ECM) has gained considerable attention for its promise in pathogenic targeting and predictive value.

**Fig. 1:**
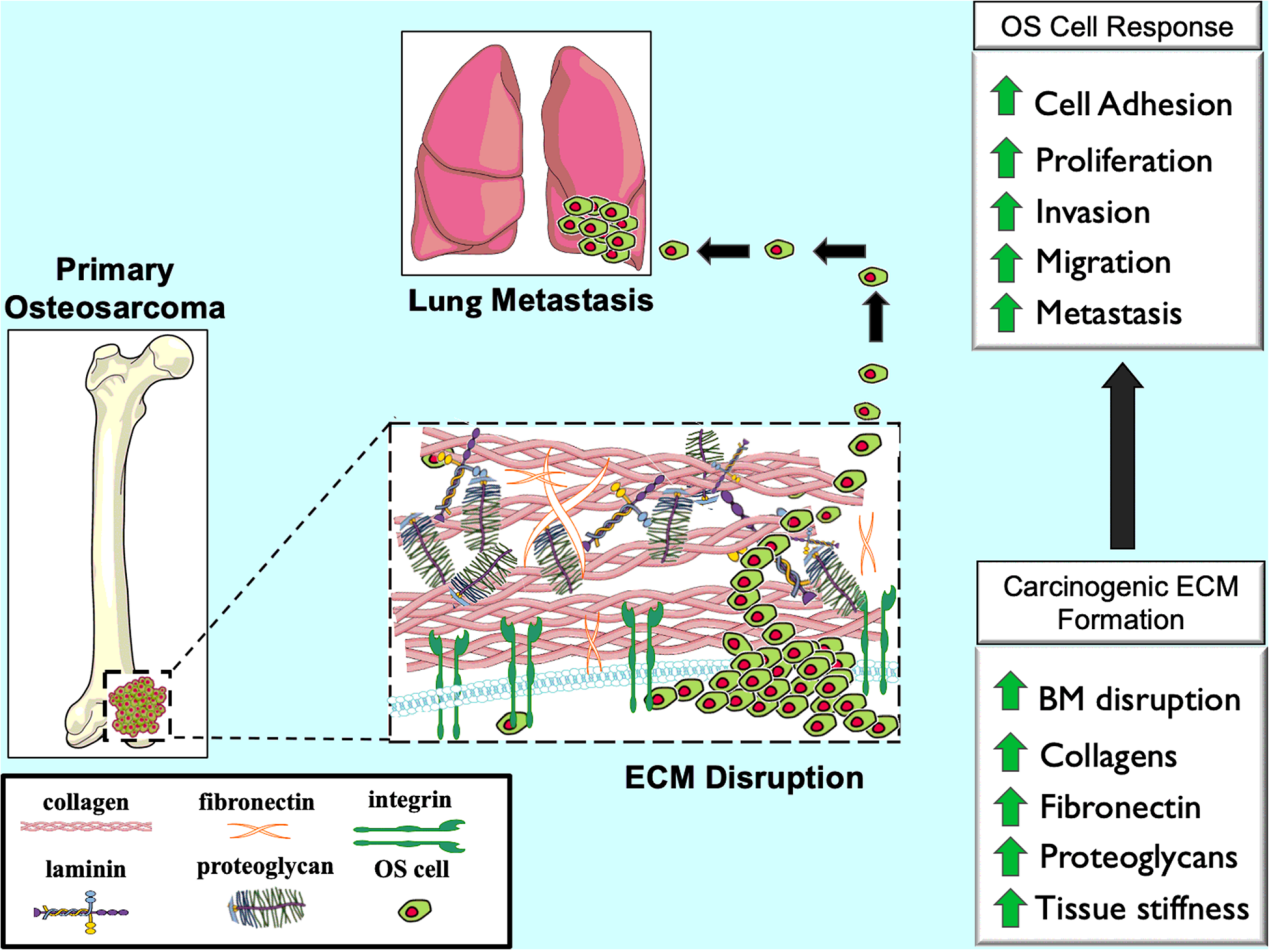
ECM changes in OS progression and metastasis. The primary components of ECM in normal bone are significantly changed in osteosarcoma (OS). Due to activated fibroblasts, cancer cells, collagen deposition, fibronectin, and other ECM components, ECM production is dramatically increased which results in a stiffer stroma and more aggressive phenotype. The basement membrane surrounding the primary tumor site is broken down by ECM remodeling enzymes allowing for OS cells from the primary tumor to undergo hematogenous spread where they frequently seed the lung

Very recently, the tumor microenvironment (TME) has gained prominence outside of its traditional role of cellular support as a veritable contributor to cancer progression and metastasis [6]. The TME, consists of a complex arrangement of blood vessels, fibroblasts, immune cells, endothelial cells, signaling molecules, extracellular vesicles and most importantly, the ECM. The ECM forms a three-dimensional acellular network of macromolecules which provide the necessary structural and biochemical support of its cellular constituents [7–9]. In addition to its function as a supportive framework, the ECM regulates most cellular behaviors, including communication, migration, adhesion, proliferation, and differentiation [10–12]. Furthermore, when aberrant, these functions are hijacked and form a specific ECM remodeling profile that enables metastatic dissemination of cancer cells [13, 14]. These features of ECM transformation have been reported in OS development and progression, a tumor with a characteristically robust ECM [15, 16]. For OS, the generation of pathogenic osteoid matrix and other ECM components enables a supportive scaffold for rapid tumor progression [17, 18] (Fig. 1).

In this review, we summarize the most recent discoveries of ECM contribution to OS progression and metastasis. We also detail the various ECM components that have shown preclinical and clinical promise as prognostic predictors and therapeutic targets in OS.

## ECM components and their function in OS

The ECM is primarily composed of collagen, fibronectin, laminin, and proteo- glycan which shape and maintain tissue vitality [7, 19, 20]. In the pathological state of cancer, however, ECM cultivates tumorigenesis and metastasis in malignancies such as OS [21–25] (Table 1).

**Table 1:** The ECM components involved in OS

ECM protein		Expression in OS	Roles in OS	References
**Collagens**	Collagen I	Increase	Invasion and metastasis	[26]
	Collagen III	Increase	Chemotherapy resistance	[27]
	Collagen IV	Increase	Angiogenesis	[28]
	Collagen V	Increase	Adhesion	[29]
	Collagen XVIII	Decrease	Anti-angiogenesis	[30, 31]
			Cell growth and Metastasis	[32]
**Fibronectin**		Increase	Adhesion	[33–35]
			Chemotherapy resistance	[36]
			Metastasis	[37, 38]
			Invasion	[39]
**Laminins**		Increase	Adhesion	[40]
			Invasion	[41, 42]
**Proteoglycans**	Biglycan	Increase	Cell growth	[43, 44]
	Decorin	Decrease	Migration	[45]
			Cell growth	[46]
	Lumican	Increase	Cell growth	[47]
			Adhesion	[48]
	Versican	Increase	Migration and invasion	[49]
	HA	Increase	Proliferation and invasion	[50–52]
			Cell apoptosis	[53]
			Metastasis	[54, 55]

### Collagens

Collagens are the main organic components of the ECM and represents approximately 30% of the total protein mass of the human body [56]. The collagen superfamily includes 28 members, each with their own unique three polypeptide chains assembled into a final triple helix structure [57, 58]. Several collagens have been investigated in OS, including collagens I, III, IV, V, and XVIII. The exact changes these collagens undergo are of considerable interest in OS progression especially given their abundance to the OS stroma.

#### Collagen I

Collagen I is composed of two alpha 1 chains and one alpha 2 chain, which are encoded by the COL1A1 and COL1A2 genes, respectively [59]. Collagen I is a rich ECM component and found in connective tissues such as bone, tendon, and ligament [60]. Elevated concentrations of collagen I metabolites have been found in untreated OS patients’ serum [61], and supplementation with exogenous collagen I has shown to increase the synthesis and activation of MMP-2 in OS cell lines [62]. This is of interest, as MMP-2 alone has been shown to promote OS progression, invasion, and migration [26]. Additionally, MMP2 activity is significantly increased in those OS patients with poor response to chemotherapy [63].

#### Collagen III

Collagen III is composed of three identical peptide chains encoded by the COL3A1 gene and is found throughout cortical bone [64, 65]. A significantly higher level of COL3A1 mRNA expression has been observed in chemoresistant patients compared to those with a more favorable response to therapy [27]. Furthermore, overexpression of COL3A1 in methotrexate-resistant OS cell lines significantly reduces apoptosis via the activity of miR-29abc, a miRNA in the miR-29 family [27].

#### Collagen IV

Collagen IV is a heterotrimer composed of three different α chains from alpha 1 to alpha 6 [66]. These chains are encoded by the COL4A1- COL4A6 genes. Collagen IV is a major constituent of basement membranes in the ECM and is heavily involved in interaction with other cellular components [67]. In a combined culture system with a 3D OS cell line and 2D endothelial cell line, the endothelial cells formed a vascular network expressing collagen IV. These networks infiltrated the nearby tumor spheroids with tubular structures. These results support the role of collagen IV in regulating OS angiogenesis, a well-known feature of tumor proliferation [28].

#### Collagen V

Collagen V exists as an alpha1, alpha2, and alpha3 heterotrimer which are encoded by COL5A1, COL5A2, and COL5A3 genes, respectively [68]. While collagen V is a relatively minor component of the ECM, it has critical roles in matrix organization alongside collagen I [69]. Together, the deposition and cross-linking of collagen I and collagen V are the principal components of cultured OS cell ECM [70]. Collagen V is especially important in the cell contact and interactions of OS, as the peptides derived from the basic segment of the alpha 3 chain of collagen V form adhesive qualities [29].

#### Collagen XVIII

Collagen XVIII contains 10 collagenous domains encoded by the COL18A1 gene [71]. This collagen is a component of basement membranes in the ECM, with structural properties of both collagen and proteoglycan [72]. Proteolytic cleavage within the C-terminal domain of collagen XVIII releases a fragment, endostatin, with anti-angiogenic effects [73]. Endostatin is important in the progression of various tumors, including OS [74, 75]. As angiogenesis is important for OS progression and metastasis, researchers elected to analyze the effects of endostatin in an orthotopic OS model. Results were positive, as their endostatin anti-angiogenic therapy significantly reduced the postoperative progression of pulmonary metastasis [30, 31]. Another study showed a combination of endostatin with Adriamycin produced synergistic inhibition of tumor growth and pulmonary metastases in an orthotopic OS model [32].

### Fibronectin

Fibronectin is an adhesive glycoprotein of the ECM composed of two polypeptides which bind integrins, collagen, fibrin, heparin, and proteoglycans [76, 77]. It forms a multidimensional fibrillar matrix with partial control of cell adhesion, migration, and differentiation [78–80]. Abundant expression of fibronectin is apparent in OS cell lines [81]. The heparin-binding domain of fibronectin affects cell adhesion and spreading of OS cells by cooperating with the central cell-binding domain of fibronectin [33]. Significant upregulation of fibronectin had been detected in chemo- resistant OS cell lines [36].

Fibronectin displays various functional motifs that interact with integrins, which are the most common transmembrane receptors and regulate its function [82–84]. The integrin structure is formed by heterodimers of α and β subunits which penetrate the cell membrane and form several cytoplasmic domains [85]. The binding of fibronectin with integrins represents a crucial step in OS progression and metastasis [37, 38]. In a recent in vitro work, integrins were shown to be involved not only in cell adhesion but also in the binding and assembly of exogenous fibronectin [34]. Selective down-regulation of integrins resulted in the decreased deposition of fibronectin within the ECM and subsequently reduced overall OS cell spreading and adhesion [39]. Conversely, upregulation of integrins enhanced adhesiveness of OS cells to fibronectin [35].

### Laminins

Laminins are components of the basement membrane in ECM and are constructed of heterotrimeric glycoproteins with alpha, beta, and gamma chain subunits [86, 87]. They interact with their respective cancer cell receptors whereby they promote angiogenesis, invasion, and metastasis [88]. Laminins have demonstrated to enhance cell adhesion in OS cell lines [40], with high laminin-adherent OS cells showing notably more invasiveness than their low laminin-adherent counterparts [41]. In a work which implemented a 3D OS cell line model, a matrix supplemented with laminin led to an increased invasion of OS cells into the surrounding acellular bone marrow environment [42].

### Proteoglycans

Proteoglycans are heavily dispersed throughout the ECM and are composed of glycosylated proteins with a protein core and covalently attached glycosaminoglycan (GAG) chains [89, 90]. The GAGs are major regulators of metastasis in various cancers [91–93]. Hyaluronic acid (HA), also known as hyaluronan or hyaluronate, is another macromolecule that belongs to the GAG family. HA is abundant in most tissues and has unique properties as a result of its variable covalent bonding and core proteins [90, 94]. And although HA is not a true proteoglycan, it possesses similar biological functions. It is synthesized on the cytoplasmic membrane and is directly secreted into the ECM [95]. Based on the core protein and GAG chain properties, proteoglycans are classified into one of three groups, including small leucine-rich proteoglycans (SLRPs), modular proteoglycans, and cell-surface proteoglycans [90]. Overall, these unique variants have roles in ECM communication, tumor angiogenesis, progression, and metastasis [96, 97].

#### SLRPs

SLRPs have relatively short protein cores with a central domain of leucine-rich repeats [98]. The SLRP family is divided into five classes according to structure and includes classes I to V [99, 100]. Functionally, these proteins regulate ECM organization and cell behavior [101]. Of the SLRP family members, biglycan, decorin, and lumican have been investigated in OS.

Biglycan is a class I SLRP encoded by the BGN gene which promotes proliferation and differentiation in OS cells [43, 102]. A mechanistic study has revealed biglycan enhances OS cell growth through the low-density lipoprotein receptor- related protein 6 /β-catenin/IGF-I receptor signaling pathway [44].

Decorin is another class I SLRP and a small pericellular matrix proteoglycan with a structure closely related to biglycan. That is where their similarities end, however, as its presence negatively correlates with oncogenesis. Decorin inhibits OS cell migration through its glycosaminoglycan side chains [45, 103]. Ectopic expression of decorin significantly decreased OS cell growth through the induction of cyclin-dependent kinase inhibitor P21 [46].

Lumican is a class II SLRPs and encoded by the LUM gene [104]. It positively correlates with OS cell differentiation and inversely correlated with growth [47]. In a subsequent study, lumican was shown to regulate OS cell adhesion by modulating transforming growth factor beta-2 activity [48].

#### Modular proteoglycans

Modular proteoglycans are multidomain motif proteins with a highly glycosylated structure [105]. They are subdivided into families of HA-binding, lectin-binding, and non-HA-binding proteoglycans [90, 96]. The four proteoglycans versican, aggrecan, neurocan, and brevican constitute the family of HA-binding proteoglycans [106]. Versican is notable for its ability to regulate cellular processes including adhesion, proliferation, apoptosis, and invasion [107, 108]. High expression of versican has been found in OS tissues relative to normal tissues [49]. Its expression is up-regulated by transforming growth factor-beta 1 (TGFß1), which leads to enhanced OS cell migration and invasion [49].

#### Ha

As previously stated, HAs have similar functions to proteoglycans [94]. They exist in all tissues and are abundant in bone [109]. In addition to their structural importance, HAs have strong roles in cancer cell differentiation, proliferation, and migration when aberrantly expressed [94]. HA promotes OS cell proliferation and invasion by initiating intracellular signal transduction [50]. In a work where HA accumulation was selectively inhibited, there was a substantial decrease in OS cell proliferation, motility, and invasiveness [51]. The inhibition of HA can also reduce cell viability and induce apoptosis in OS cells [53]. At the microscopic level, cells interact with HA through cell surface receptors, which initiates their actions. The cluster of differentiation 44 (CD44) is a well-known cell membrane receptor for HA. When HA is bound to CD44, it regulates cell-cell interactions, cell adhesion, and migration [110]. The HA-CD44 pathway increases tumor aggression and drug resistance as well as influencing the cancer stem cell phenotype through promoting stem-cell gene expression, progression, and metastasis [111]. Of note, the expression of CD44 is significantly higher in metastatic and recurrent OS patient tumor specimens compared to primary tumor tissues [54]. Therapeutically, the proliferation and spheroid formation of OS cells is inhibited in 3-D culture when CD44 is silenced [52]. In an orthotopic mouse model of OS, injection with CD44 overexpressing OS cells resulted in increased primary tumor formation and lung metastasis, which was dependent on the HA to CD44 interaction [55].

## Signaling pathways responsible for ECM remodeling in OS

**Fig. 2:**
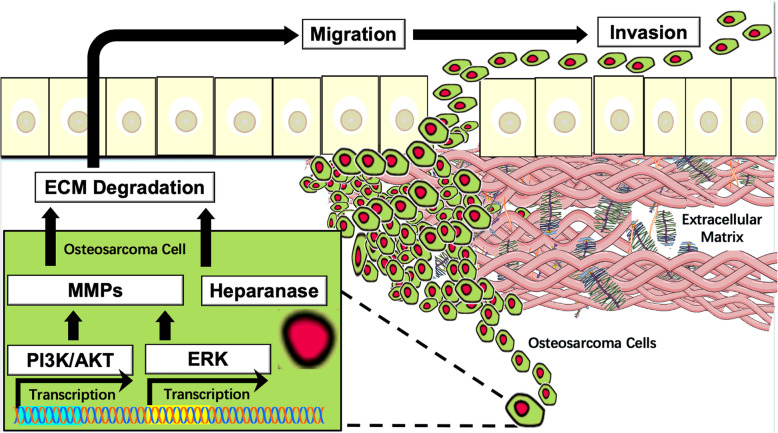
Schematic of signaling pathways involved the ECM remodeling in OS. The ECM is dynamically remodeled by multiple proteases and signaling pathways. In OS, MMP-2, MMP-9, and MMP-13 function via PI3K/Akt and ERK associated signaling pathways. Heparanase also participates in ECM remodeling, as it cleaves the ECM by heparan sulfate degradation, thus promoting OS cell invasion and metastasis

The function of ECM is derived from its diverse composition of macromolecules, proteases, inhibitors, and their respective downstream signaling pathways [112]. Within the ECM of OS, matrix metalloproteinases (MMPs) and heparinases regulate several pathways responsible for progression and metastasis (Fig. 2).

### MMPs

MMPs are proteolytic enzymes that degrade surrounding ECM components, release active growth factors, and promote tumor angiogenesis [113]. Elevated levels of MMP-2, MMP-9, and MMP-13 exist in OS (Fig. 2) and contribute to cell migration, invasion, and metastasis. The upstream PI3K/Akt signaling pathway promotes the expression of MMP-2 and MMP-9, thus degrading ECM and enabling OS cell invasion and metastasis [114, 115]. The extracellular signal-regulated kinase (ERK) signaling pathway also upregulates MMP-2 and MMP-9 and migratory activity of OS cells [116, 117]. Another MMP, MMP-13, causes turnover of ECM collagens and proteoglycans and directly correlates with OS progression. In one recent study, plasminogen activator inhibitor-1 was shown to upregulate the expression of MMP-13 and promote invasion and lung metastasis in an OS mouse model [118].

### Heparanase

Heparanase is an endo-β-D-glucuronidase that cleaves heparan sulfate chains in the ECM, thus releasing heparan sulfate-binding angiogenic factors and allowing for tumor cell migration, invasion, and metastasis [119, 120] (Fig. 2). Previous works have shown the down-regulation of heparanase significantly reduces OS cell proliferation and invasion [121, 122]. Additionally, OS patient tissues with more heparanase correlate with higher microvessel density and rates of pulmonary metastasis [122].

## ECM components as prognostic biomarkers in OS

Although the adoption of neoadjuvant chemotherapy in OS has significantly improved patient survival since its implementation several decades ago, outcomes have since plateaued. The personalized and immunotherapies that have shown great promise in several cancers have had less favorable results for OS, likely due in part to its heterogeneity between patients. There is, therefore, an urgent need for prognostic biomarkers which allow for the delineation of patients according to their unique tumor microenvironments and response patterns, so that their therapeutic regimens can be tailored accordingly. In response, there has been an expansion of works investigating the components of the ECM, some of which have been found to play vital roles in cancer progression, metastasis, and clinical outcomes [123–125]. An emergence of clinical data has revealed various collagens to correlate with the clinical stage, metastasis, and outcomes [126]. The correlation between ECM makeup with clinical stage and prognosis in OS are summarized (Table 2). Several noteworthy examples exist, including the expression of collagen triple helix repeat containing 1 (CTHRC1) protein in OS. It has significantly higher expression compared to adjacent normal tissue controls, and predicts a poor prognosis of OS patients [127]. Functionally, CTHRC1 is a secretory protein known to regulate vascular remodeling and bone formation [128]. A collagen I (COL1A1) polymorphism is associated with OS susceptibility and death [129]. Fibronectin is overexpressed in OS specimens compared to osteochondroma as well as other tissues [130, 131]. Additionally, overexpression of fibronectin in OS tissues is associated with a poorer chemo- therapeutic response, distant metastasis, and shorter overall survival [130, 131]. In short, these works support high fibronectin expression as an underlying mechanism of aggressive clinical behavior in OS. A higher level of CD44 expression in OS tissues is apparent in patients with shorter survival and those with an unfavorable response to neoadjuvant chemotherapy [54]. Furthermore, CD44 expression is predictive of poor survival, metastasis, recurrence, and drug resistance in patients with OS [132, 133].

**Table 2:** ECM as prognostic predictors in OS

ECM components		Expression in OS	Prognostic value	References
**Collagens**	CTHRC1	High	Shorter survival time	[116]
	COL1A1	High	Shorter survival time	[118]
**Fibronectin**		High	Metastasis, poor response to chemotherapy, and shorter survival time	[119, 120]
**Proteoglycans**	CD44	High	Poor response to chemotherapy	[54]
			Metastasis, recurrence and shorter survival time	[121, 122]

## ECM components as potential therapeutic targets in OS

The ECM is pivotal in OS pathogenesis, especially in tumor cell migration and invasion. Targeting the regulatory and responsible molecules within the ECM has thus been explored as a novel strategy for OS treatment (Table 3).

**Table 3:** ECM as therapeutic targets in OS

Therapeutic target		Functions	References
**Collagens**	COL3A1	Methotrexate resistance, apoptosis	[27]
	Tumstatin	Cell proliferation, apoptosis	[124]
	Endostatin	Metastases	[125]
**Fibronectin**	Fibronectin	Doxorubicin sensitivity	[36]
	Integrins	Cell proliferation, metastasis	[129]
**Proteoglycans**	Decorin	Cell invasion, metastasis	[130]
	CD44	Doxorubicin sensitivity	[54]

### Collagen targets

Overexpression of COL3A1 can decrease apoptosis and promote methotrexate resistance in OS cell lines. The precise targeting of COL3A1 is therefore a promising and personalized strategy for overcoming methotrexate resistance in candidate OS tumors [27]. The antiangiogenic protein fragment tumstatin, which is cleaved from collagen, is the non-collagenous domain of the alpha 3 chain in collagen IV shown to inhibit cell proliferation and induce cell apoptosis in OS cell lines [134]. Mechanistically, this occurs through the phosphorylation of p65NF-κB and its subsequent nuclear translocation [135]. Tumstatin has therefore become of interest in the treatment of OS [135]. Endostatin combined with other chemotherapy has been evaluated in an OS clinical trial, with results showing a significant reduction in angiogenesis, metastasis, and an increased event-free survival rate [136]. Overall, endostatin-targeting angiogenesis-based therapy has yielded positive results for OS patients at the clinical trial level.

### Fibronectin targets

The fibronectin and integrin families within the ECM regulate a diverse array of cellular functions crucial for proliferation, progression, and metastasis [137]. Therapeutically, fibronectin inhibition greatly increases OS sensitivity to doxorubicin in vitro. Similarly, fibronectin knockdown decreases the tumor growth rate and can even resensitize OS to doxorubicin in orthotopic OS models [36]. Consequently, targeting fibronectin has become a promising treatment for doxorubicin-resistant OS [36]. As the main receptor of fibronectin, integrins are also proposed targets of cancer treatment. Several studies have shown inhibition of integrin or its downstream effectors to block many of the major hallmarks of cancer [137–139]. Additionally, selective knockdown of integrins significantly inhibits OS growth and lung metastasis, and an exogenous reintroduction of integrins can restore cell proliferation and lung metastasis in xenograft models of OS [140]. As pulmonary metastasis is the major cause of patient death in OS, these findings are especially promising and warrant future works.

### Proteoglycans targets

In a murine OS model, significantly fewer pulmonary metastases and longer survival times were observed in mice treated with decorin, a matrix proteoglycan. The works of these investigations support decorin as a potential therapeutic target in the prevention of lung metastasis in OS [141]. As previously described, CD44 is important in OS progression. Furthermore, it is the direct target of miR-199a-3p, which is a significantly downregulated miRNA in OS [142, 143]. As a therapeutic strategy, overexpression of miR-199a-3p significantly inhibits CD44 expression in OS cell lines, with transfection also increasing chemosensitivity. Taken together, these results support targeting CD44 to reduce pulmonary metastasis and increase OS clinical outcomes [54].

## Conclusion and future perspectives

In addition to is physiologic importance in structural and biochemical support, the ECM has gained increased recognition for its carcinogenic roles, including in the progression and metastasis of OS. The various components of the ECM including collagens, fibronectin, laminins, and proteoglycans may contribute to OS progression and metastasis through distinct and intertwining mechanisms. It is therefore important to further study and validate the ECM components, cellular receptors, and associated signaling pathways in OS synergistically and as components of the primary tumor tissue. Novel culture systems will be especially important in this endeavor, as resembling the in vivo tumor microenvironment with in vitro customizability, such as with 3D cell culture, will be necessary to accurately model extracellular matrix and growth. Overall, the ECM components have shown promise as clinical biomarkers and therapeutic targets in OS, and warrant a continued evaluation in preclinical models as well as future clinical trials.

## Data Availability

Not applicable.

## Abbreviations

OS: Osteosarcoma
TME: Tumor microenvironment
ECM: Extracellular matrix
GAG: Glycosaminoglycan
HA: Hyaluronic acid
SLRPs: Small leucine-rich proteoglycans
CTHRC1: Collagen triple helix repeat containing 1
MMPs: Matrix metalloproteinases
